# A Large Language Model–Powered Multiagent Framework Emulating Standardized Patients in Clinical Communication Skills Training: Development and Evaluation Study

**DOI:** 10.2196/84747

**Published:** 2026-06-04

**Authors:** Yufei Qu, Xiaowei Xu, Yunzi Long, Yijie Wang, Jiao Li, Xudong Lu

**Affiliations:** 1 College of Biomedical Engineering and Instrument Science, Zhejiang University, No. 38 Zheda Road, Hangzhou, 310058, China; 2 Institute of Medical Information/Library, Chinese Academy of Medical Sciences and Peking Union Medical College, Beijing, China; 3 Department of Cariology and Endodontology, Peking University School and Hospital of Stomatology and National Center for Stomatology and National Clinical Research Center for Oral Diseases and National Engineering Research Center of Oral Biomaterials and Digital Medical Devices and Beijing Key Laboratory, Beijing, China; 4 Department of General Dentistry II, Peking University School and Hospital of Stomatology, Beijing, China; 5 Hangzhou Joyrun Medical Science and Technology co., LTD, Hangzhou, China

**Keywords:** virtual patient, large language models, multiagent, medical education, communication skills

## Abstract

**Background:**

Effective clinical communication is essential for medical practice, with standardized patients (SPs) being a reliable standard training method despite resource limitations. While large language models (LLMs) show strong role-playing abilities, current virtual patients (VPs) based on single LLMs face fidelity and interaction challenges. Recent advances in multiagent frameworks, which have demonstrated considerable potential in handling complex tasks, offer a new perspective for creating VPs in medical education.

**Objective:**

This study aimed to develop and evaluate a novel multiagent VP framework that simulates SPs through a collaborative agent design, thereby enhancing human-like fidelity and interaction performance in clinical communication training–oriented VP simulation.

**Methods:**

Our multiagent framework constructed 5 specialized subagents by simulating the functional partitioning of brain regions, collaboratively simulating the entire process, from case reception to interactive consultation scenarios, designed for medical students. To enhance the interaction performance of VPs, we incorporated retrieval-augmented technology, while deep character reasoning was used to improve response richness and realism. We evaluated the proposed framework through a 2-phase experiment in which the metrics of response quality, role-playing performance, interaction efficiency, information accumulation, and perceived educational utility were applied consistently: first, to compare different base models, and second, to benchmark the complete framework against a single-LLM baseline.

**Results:**

The multiagent framework outperformed single-LLM baselines across multiple evaluation settings, achieving high information accuracy and role-playing scores under standardized dialogue conditions. Specifically, the GPT-4o–based implementation achieved peak factual consistency of 0.769 (SD 0.04), while all configurations maintained >94% clinical accuracy. The Qwen3-32B–based framework achieved the lowest misleading rate of 1.28% (SD 1.20), compared to 4.72% (SD 1.53%) for single-LLM scoring. In assessments using standard dialogue scripts, the Qwen3-32B–based framework attained the highest role-playing competency score of 39.67 (SD 0.71) and received high expert praise. However, limited discriminative power against specific leading questions on low-quality inquiries indicated that while these findings specifically establish high fidelity under structured conditions, further adaptation is required for authentic student interactions. Interaction efficiency remained practical with acceptable latency (~3 s) based on Qwen3-32B while maintaining a stable information pace during multiturn dialogues. Furthermore, a preliminary exploration of factual consistency and role-playing ability across 5 clinical departments demonstrated potential scalability.

**Conclusions:**

The multiagent framework offers a viable simulation of SPs through the coordinated interaction of multiple LLM-based agents. This approach enhances the performance of VP simulation, providing a customizable and scalable solution for medical communication training, without compromising patient confidentiality. The framework holds substantial potential for advancing medical education approaches.

## Introduction

### Background

In the medical field, proficient clinical communication skills, encompassing medical history taking, physical examination, diagnosis, and decision-making processes, constitute fundamental competencies for clinical practice [1]. Strong communication skills enable physicians to obtain accurate information during diagnosis and treatment, thereby increasing diagnostic quality [2] and establishing effective physician-patient relationships [3]. These demands place increasing emphasis on the development of medical students’ clinical abilities.

To enhance clinical communication skills, medical education uses a variety of approaches, such as didactic lectures [4], feedback [5], standard curriculum [6], role-play [7], and standardized patients (SPs) [8], as well as digital strategies [9], including online modules, virtual patient (VP) simulations, and blended digital education. Among these, SPs, trained actors simulating real patients, are recognized as one of the most effective and widely adopted methods [10]. However, SP-based training faces significant challenges, including high resource requirements for recruitment, training, and scenario design [11], as well as SPs’ anxiety and fatigue. Owing to these limitations, traditional teaching methods are still predominantly used in instruction [12], which remain insufficient for strengthening clinical competencies [13].

### Prior Work

Recent advances in large language models (LLMs) have unlocked unprecedented capabilities in contextual interaction [14], dynamic role-playing [15], and clinical reasoning [16], establishing a technological foundation for developing VPs as scalable alternatives to SPs [17]. The construction of patient simulators has been extensively explored for both training and evaluating clinical LLMs. In the training domain, systems such as AMIE [18] leverage patient agents derived from structured literature profiles to optimize performance via self-play and chain-of-reasoning, which prioritize authenticity, relevance, and fidelity to generate robust fine-tuning consultation dialogue datasets. In the evaluation domain, patient simulators serve as benchmarking frameworks to assess diagnostic accuracy in simulated clinical environments [19,20]. Notably, the Baichuan framework [21] enhances the patient simulator reliability by integrating rule-based judgment, dynamic prompting, and real-time correction modules to ensure output validity. Furthermore, research on VPs tailored for student education primarily prioritizes the responsiveness to medical inquiries and the interactive conversational experience. Initial explorations [22-24] using prompt engineering and visual augmentation have demonstrated the feasibility of LLM-based VPs. However, single-LLM implementations still suffer from limited fidelity in persona simulation [25] and persistent hallucination issues [26]. Beyond conventional optimization strategies such as fine-tuning [15] and reinforcement learning [27], recent studies increasingly explored multiagent architectures, which have proposed various organizational frameworks for multiagent systems (MAS), including flat, hierarchical, holonic, coalition-based, team-oriented, matrix, and congregation structures [28], enhancing LLM performance through agent collaboration and complex task decomposition [29].

Although research on multiagent VPs remains limited, notable efforts include SimPatient, which uses 3 specialized subagents (patient response generation, behavior coding, and cognitive modeling) to strengthen clinical authenticity in patient interactions [30], and Yu et al [31], who introduced a retrieval-augmented methodology based on a knowledge graph, enabling subagents to collaboratively perform retrieval tasks and improve response accuracy in clinical reporting. Despite these advances, current solutions remain inadequate for large-scale educational adoption due to insufficient system flexibility [11] and lack of robust evaluation metrics, particularly those assessing role-playing fidelity [32]. This underscores the need for further research on VP systems tailored for clinical communication training, with a focus on accurate clinical information delivery, anthropomorphic authenticity, and scalability.

### Goal of This Study

The aim of this study was to develop and evaluate a multiagent VP framework for clinical communication training. The proposed framework is designed to improve simulation fidelity and validity, interaction performance, and scalability of VP simulations and to systematically examine whether a multiagent architecture provides advantages over single-LLM approaches in SP emulation.

## Methods

### Overall Study Design

**Figure 1.**
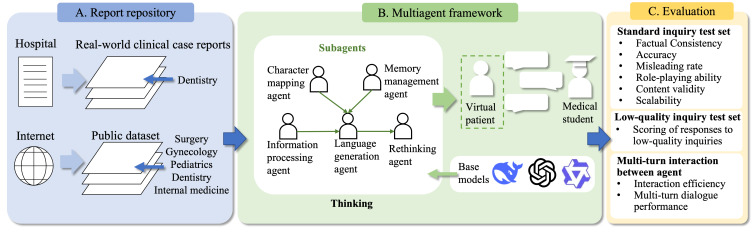
Overview of the study design, including the report repository, multiagent framework, and evaluation process.

This study aimed to develop and evaluate a multiagent framework for VPs (Figure 1). The research pipeline comprised the following steps. First, clinical case reports were collected from real-world medical records and publicly available datasets to build the VP system. Subsequently, a multiagent framework with 5 subagents was designed to simulate SPs in clinical communication training scenarios, using the collected case reports. Finally, a comprehensive evaluation was conducted to select a relatively well-rounded base model for the framework and to assess the performance of the MAS in comparison with a single-LLM approach.

### Ethical Considerations

Ethical approval for the study was granted by the Biomedical Institutional Review Board of the Peking University School and Hospital of Stomatology (PKUSSIRB-2025115200). Participants were informed about their involvement in this research, and all procedures strictly adhered to ethical standards outlined in the Declaration of Helsinki.

### Clinical Case Repository

Our clinical case repository included real-world cases and public medical datasets: (1) the CMB-Chinese Medical Benchmark dataset [33], which consists of 74 complex medical consultation cases from multiple departments, containing chief complaints, present illness, medical history, and examination results, all with personal identifiers removed; and (2) 40 real-world dental cases collected from Peking University School and Hospital of Stomatology, modified to include standardized information such as chief complaints, present illness, medical history, examination findings, and diagnoses, in line with SP teaching requirements.

### Multiagent System

On the basis of the involvement of distinct functional brain regions in the educational process of SPs (including script preparation, script learning, feedback practice, and interaction), such as the prefrontal cortex, superior frontal cortex, temporal cortex, and brainstem, we mapped these functions onto specialized subagents responsible for managing different aspects of the VP interaction. Building on this foundation, we designed subagents aimed at enhancing the role-playing performance and scalability of existing methods through the refined functional division and collaborative operation of agents. Our framework integrates 5 specialized agents to support SP simulation using a bio-inspired architecture where each agent is functionally mapped to specific brain regions: the character mapping agent embodies role traits and sustains motivational states, reflecting emotion processing in the prefrontal cortex and state maintenance in the limbic system; the memory management agent, analogous to the hippocampal-prefrontal network, enables contextual encoding, consolidation, and retrieval; the information processing agent supports cognitive flexibility and query interpretation, mimicking the adaptive functions of the prefrontal cortex and temporal lobe; the language generation agent corresponds to cerebral language comprehension and production areas to formulate coherent responses; and the rethinking agent is modeled after the integrative function within the prefrontal network, which supports performance monitoring, error detection, and behavioral adaptation.

**Figure 2.**
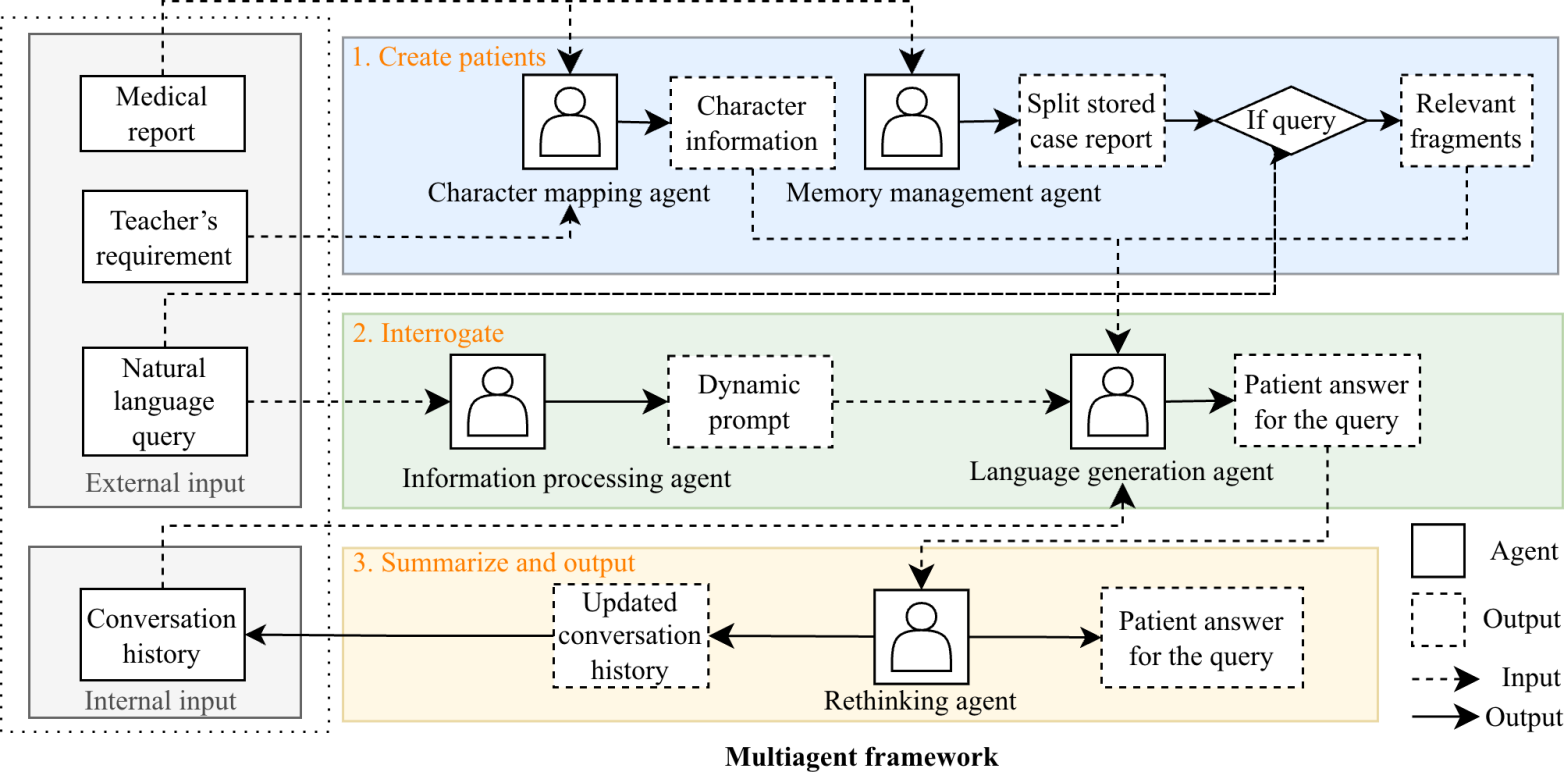
Our multiagent framework uses a 3-stage framework comprising patient construction, diagnostic reasoning, and response generation. Through simulated multiround interactions mimicking brain regions, the system (1) identifies patient information relevant to queries in case reports, (2) generates SP responses, and (3) checks the responses. The framework incorporates persistent memory mechanisms to ensure dialogue coherence across conversational turns.

Building on these preliminary results, our study proposes a cerebral cortex–inspired MAS framework designed to simulate SP clinical training behaviors, as shown in Figure 2.

The framework operates with 3 external inputs: one of the clinical case reports in the repository, natural language queries from users, and instructional requirements from medical teachers. All subagents rely on an LLM core for tasks such as retrieval, generation, and reflection. The multiagent framework has been specifically optimized for medical education. The agent-based collaborative workflow consists of (1) a character mapping agent simulating patients’ emotional responses and character traits, (2) a memory management agent storing and retrieving case information during consultation, (3) an information processing agent evaluating students’ diagnostic questions and generating appropriate dynamic prompts, (4) a language generation agent producing clinically accurate responses, and (5) rethinking agents assessing whether responses satisfy SP standards.

**Figure 3.**
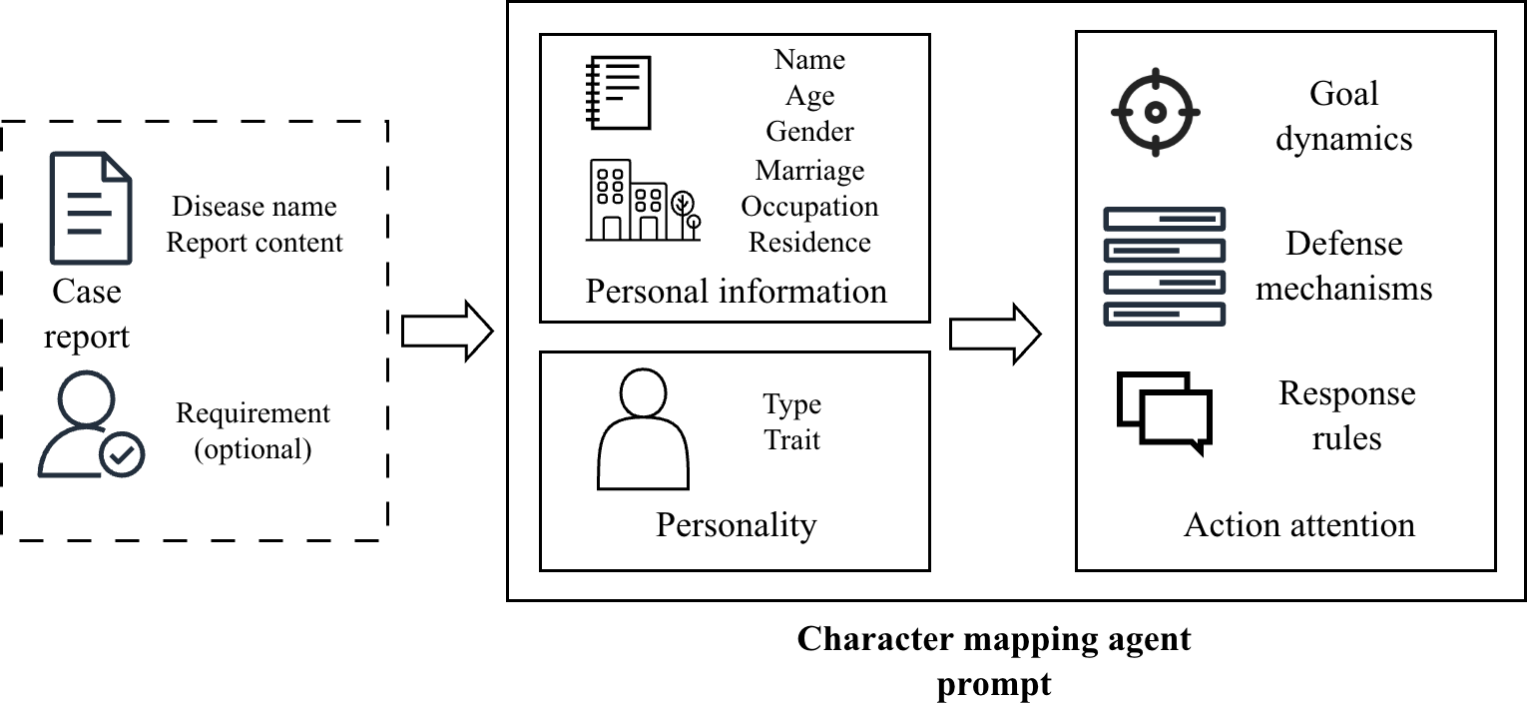
Schematic of the hierarchical prompt design for the character mapping agent.

*The character mapping agent* generates personalized VPs by drawing inspiration from neurocognitive processes, including prefrontal cortex–mediated emotional regulation, limbic system-driven motivational states, and affective responses. To protect data privacy, all patient case reports are anonymized before being processed by the LLM to synthesize multidimensional characteristic profiles. The agent uses a hierarchical prompt architecture in which the shallow instruction layer constructs personality profiles according to the Five-Factor Model [34], while concurrently processing medical records and teacher inputs to generate associated demographic markers. Specifically, teacher input refers to the patient attributes specified by the teacher, including age, personality, and communication barriers, for the generation of a customized VP. Subsequently, the deep instruction layer operationalizes these personality dimensions into explicit behavioral protocols, systematically defining latent objectives, response thresholds, and psychological defense mechanisms as specified in Figure 3.

**Figure 4.**
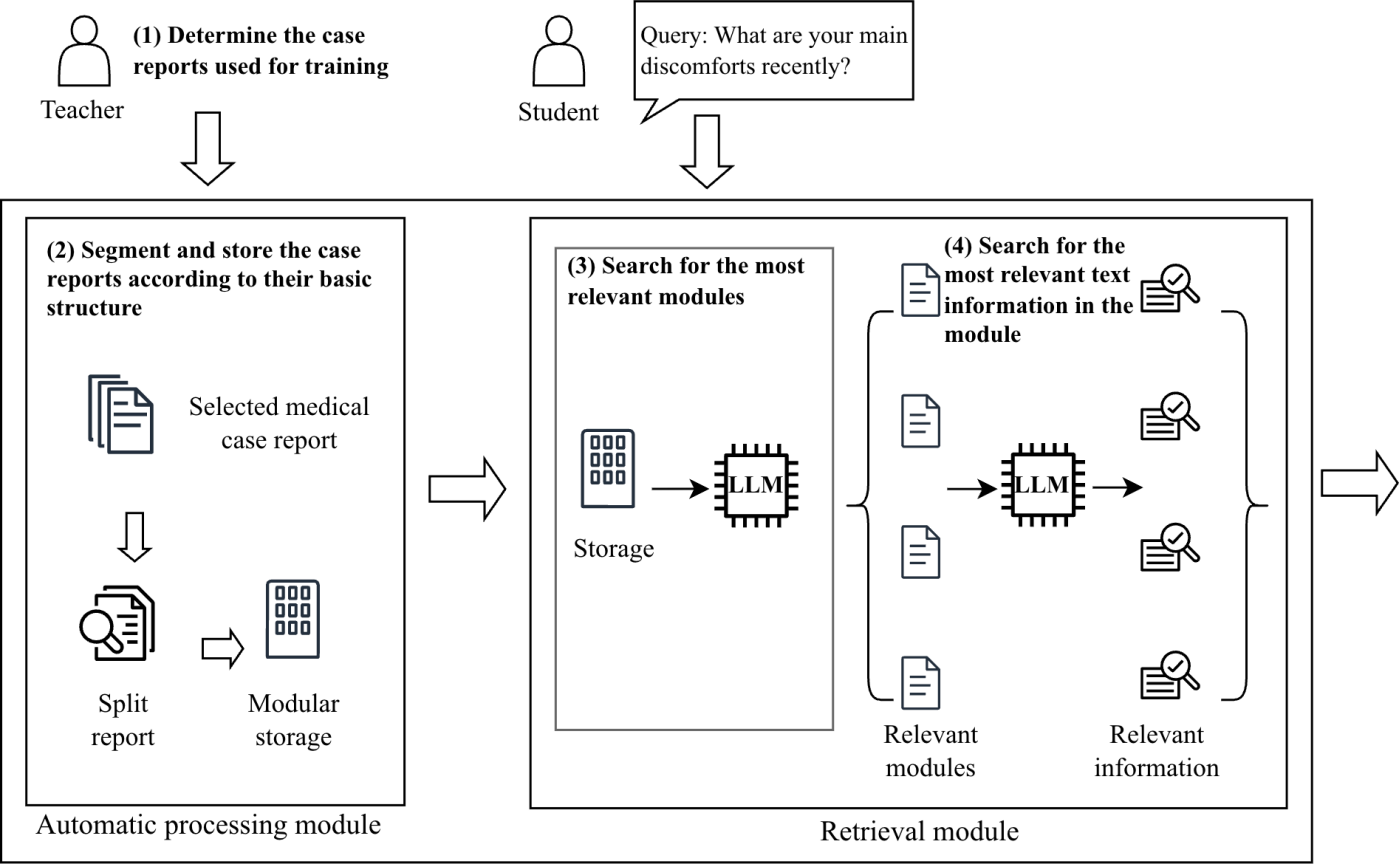
The workflow for automatic processing and retrieval of clinical case reports of the memory management agent. LLM: large language model.

*The memory management agent* uses a dual-module architecture, as shown in Figure 4. Unlike traditional retrieval-augmented generation methods, the dual-module architecture of this agent is designed to achieve retrieval enhancement by leveraging the semantic analysis capability of LLM. The automatic processing module structures clinical data into standardized medical categories, including chief complaint, present illness, past history, family history, and examination results, and then segments each category into minimal semantic units. For retrieval, the agent uses prompt engineering to guide the core LLM in performing semantic analysis and selecting the most relevant partitions. When it receives a user query, the agent then processes multithreaded parallel tasks to extract the most relevant information from each module. Finally, the agent aggregates these results to return the most relevant case report information.

**Figure 5.**
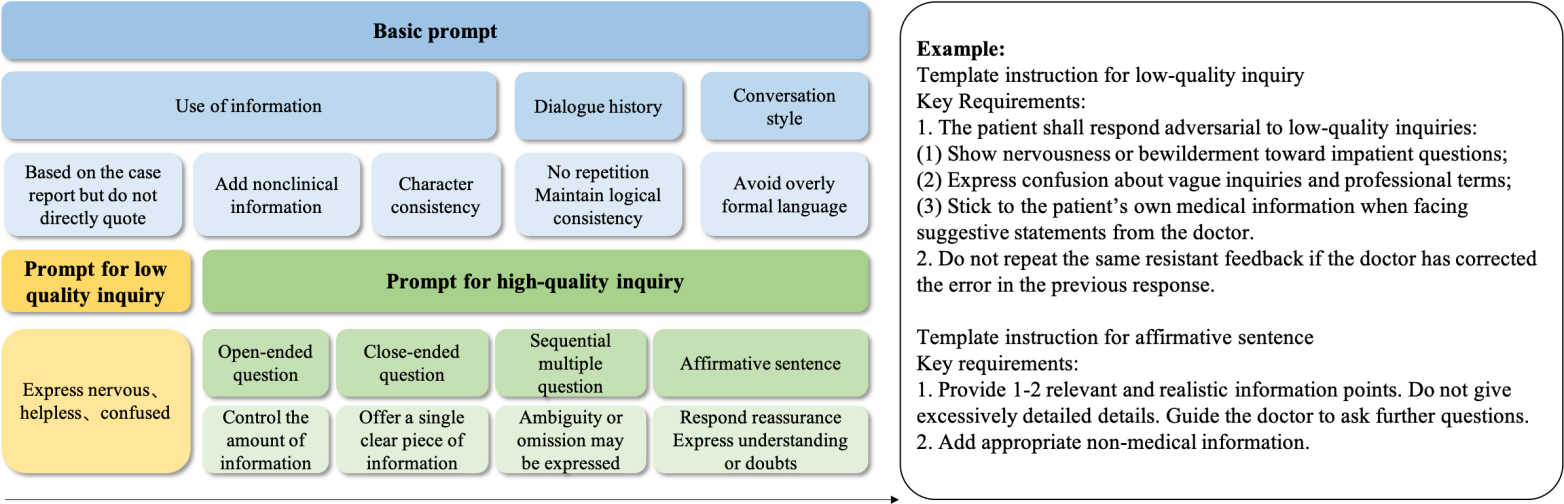
Content of dynamic prompts, including consistency prompts and variable prompts guided by the information processing agent.

*The information processing agent* functionally maps to the prefrontal-temporal cortex, handling input query analysis and semantic processing to generate responses during clinical interview simulations. Initially, the agent uses an LLM to assess the quality of a medical consultation, evaluating communication competencies, such as therapeutic rapport, verbal clarity, and proper use of medical terminology. The system then automatically classifies questioning patterns into clinically relevant categories, such as open-ended facilitation and closed-ended interrogation. Finally, the framework uses these analytical labels to dynamically select appropriate response strategies to guide the language generation agent, as detailed in Figure 5.

*The language generation agent* generates replies by integrating inputs from previous modules, including the patient’s personality profile, medical records, and the current query context, all while keeping track of the conversation’s memory state.

*The rethinking agent* corresponds to the monitoring functions of the prefrontal network, implementing a quality control mechanism to evaluate generated responses against SP requirements. Its key validation criteria include (1) avoidance of physician query repetition, (2) maintenance of patient-appropriate knowledge levels, (3) clinical relevance optimization, and (4) pedagogical alignment through information dosage control. Validated outputs are incorporated into the evolving dialogue history.

### Inquiry Test Set

To rigorously evaluate the VP, we developed both a standard inquiry test set and a low-quality inquiry test set, ensuring all generated data underwent expert verification by clinical faculty. The standard inquiry test set was constructed to assess response consistency under common conditions, where inquiries were generated via a few-shot learning paradigm. In the generation process, standard dialogue scripts manually authored by experienced medical teachers served as prompts to guide the GPT-4 (OpenAI) in generating standardized inquiries derived from clinical case reports. In contrast, to evaluate system robustness against novice errors, we developed the low-quality inquiry test set using a zero-shot learning approach. Drawing on prior research [35-37], we identified 5 prevalent and empirically testable error types, such as vague inquiries and terminology stacking, as detailed in Table 1. GPT-4 was prompted to generate flawed inquiry samples based strictly on these error definitions across 3 randomly selected dental cases, producing 3 distinct examples per category to ensure comprehensive coverage and validity.

**Table 1.** Example of low-quality inquiry.

Type	Description	Example
Vague and ineffective	Characterized by unfocused or overly broad questioning that fails to elicit specific, clinically relevant details	When you had scaling done at the outside hospital four years ago, did the doctor use an ultrasonic scaler or manual curettage?
Terminology stacking	The excessive use of complex medical jargon without adequate lay explanation	Has this tooth previously undergone root canal filling or pulp mummification therapy?
Rigid template application	A mechanical adherence to standard history-taking protocols, often resulting in irrelevant or redundant lines of questioning	I see your medical record notes atrial fibrillation. So, did you suffer from rheumatic fever as a child? Is there a family history of cardiac issues?
Leading questions	The phrasing of inquiries in a manner that suggests a specific answer or introduces bias	When you were in severe pain two days ago, drinking cold water made it hurt, right? This is important, so think carefully—it must have been painful, correct?
Lack of humanistic care	Neglecting the patient’s emotional state, anxiety, or need for empathy, resulting in insufficient doctor-patient rapport	Oh my, your face is swollen asymmetrically. It looks crooked... actually, it looks quite frightening.

### Evaluation

Our evaluation was conducted in 2 phases. In the first phase, we systematically evaluated 3 representative LLMs: Qwen3-32B (Alibaba Cloud) [38], DeepSeek-V3 (DeepSeek-AI) [39], and GPT-4o (OpenAI) [40], as potential foundational models for our multiagent framework. Notably, the reasoning mode of Qwen3-32B was disabled for both the multiagent framework and the single-LLM baseline. This setting was constrained by the latency of real-time interactions. Preliminary tests indicated that enabling the reasoning mode resulted in excessive response latency averaging more than 18 seconds, which is unacceptable for practical application.

We evaluated the dialogue transcripts obtained by applying the inquiry test set to the VPs, constructed from 5 specialized clinical case reports, including internal medicine, surgery, gynecology, pediatrics, and dentistry. A comprehensive performance evaluation was conducted using the metrics detailed in Table 2. First, we assessed factual consistency using text similarity metrics evaluated on cases from 5 medical specialties based on the standard inquiry test set. We adopted a human-machine collaboration approach to systematically score other metrics. And then, 5 attending physicians from Peking University School and Hospital of Stomatology were invited to conduct evaluations using a rating scale. Specifically, accuracy rate, misleading rate, and perceived educational utility were assessed using the standard inquiry test set, while the role-playing ability was evaluated using both the standard and low-quality inquiry test sets. Physicians assessed the VPs constructed based on dental cases. To ensure objectivity, a blinded evaluation was enforced in which experts were blinded to the underlying model architecture, and model outputs were presented in a randomized order to eliminate sequence bias. Furthermore, all evaluations were conducted anonymously to preserve the integrity of the assessment process. Meanwhile, we compared these evaluations with the results generated by GPT-4, a widely used LLM-as-judge [41], to verify the reliability of LLM-based assessments. Subsequently, we expanded the cases to 4 other specialties, conducted assessments using the LLM-as-judge method, and analyzed the scalability of our framework. Note that establishing comprehensive scalability requires further validation through assessments by experts from different departments. Finally, we evaluated the interaction efficiency and multiturn dialogue performance to observe the VP’s performance during realistic multiround interactions.

**Table 2.** Evaluation dimension.

Evaluation dimension	Evaluation by	Description of value	Metrics
Factual consistency	Researchers	Complementary measures to validate the delivery of essential educational information	Similarity metrics
Accuracy	Medical experts	Complementary measures to validate the delivery of essential educational information	Average score of scale
Misleading rate	Medical experts	Detect personality logical inconsistencies	Average score of scale
Scalability	Researchers and LLM^a^	Evaluate the scalability of using cases from different departments	Factual consistency and role-playing ability score
Role-playing ability	Medical experts and LLM	Verify the authenticity of the patient persona	Role-playing score evaluated by standard inquiries and scoring of responses to low-quality inquiries
Perceived educational utility	Medical experts	Assess the VP^b^’s perceived usability	Average score of scale
Interaction efficiency	Researchers	Characterized the practical usability and output stability of the multiagent system	Average response time and average token counts
Multiturn dialogue performance	Researchers	Characterized the practical usability and output stability of the multiagent system	Average dialogue turns, response length, and information accumulation curve

a LLM: large language model.

b VP: virtual patient.

To further investigate the performance of our multiagent framework in SP simulation, we conducted a second-phase experiment. This subsequent evaluation selected the best-performing LLM for our framework from the first phase and compared our multiagent framework against a single-LLM baseline using identical assessment metrics. The single-LLM baseline used identical instructor requirements and user inputs as the multiagent framework, including teacher requirements for patient profile and natural language query, with detailed prompts provided in Multimedia Appendix 1. For each session, it retrieved the corresponding disease case report to formulate its responses. The controlled comparison specifically examined the enhancement effects of the multiagent framework on LLM-based role-playing ability. Detailed descriptions of each evaluation metric are provided below.

Factual consistency is defined as the faithfulness of the disease information in the response to the original case report details. We assessed factual consistency by segmenting medical records into minimal fact units and measuring their semantic similarity with VP responses to the standard inquiry test set using the bge-small-zh-v1.5 model (Beijing Academy of Artificial Intelligence) [42]. Case scalability was then statistically analyzed using statistical approaches, including the coefficient of variation (CV). This assessment verifies whether response contents are substantiated by evidence from medical records, thereby preventing hallucinations or inaccuracies.

Accuracy rate was assessed by measuring the framework’s ability to generate clinically correct responses based on clinical case reports. Experts primarily assessed whether the responses to the standard inquiry test set contained logical errors or clinical inconsistencies and performed binary evaluations to calculate the accuracy rate as the percentage of correct responses relative to the total number of dialogues. Misleading rates were similarly determined through binary expert scoring, defined as the proportion of medically inaccurate responses based on information not derived from clinical case reports in the total dialogue set. The SP simulations incorporated both case-specific medical information and artificially generated patient characteristics, including occupation, age, and lifestyle factors. These additional features enhanced simulation realism but may compromise the correctness of the responses, potentially leading to misleading information.

The assessment of role-playing ability and perceived educational utility, based on the standard inquiry test set, used a modified 5-point Likert scale [43], covering 8 performance dimensions as presented in Table 3. Perceived educational utility was evaluated by physicians based on the suitability of dialogues for direct use as SP responses in clinical teaching. A machine-expert evaluation approach was adopted: medical physicians independently assessed a subset of dialogues, providing Likert scores for role-playing ability and perceived educational utility scores, while GPT-4 evaluated role-playing ability using the same scale criteria. Simultaneously, we used the low-quality inquiry test set to interact with the VP and generated corresponding dialogue logs as an exploratory analysis. As the standardized 5-point Likert scale was not fully suitable for evaluating low-quality responses, 5 physicians were invited to conduct a binary assessment of the VP’s responses. The primary evaluation criterion was consistency with the assigned persona. Specifically, physicians judged whether the VP exhibited appropriate emotional and behavioral reactions, such as expressing skepticism, confusion, or rebuttal, when confronted with these low-quality inquiries, rather than simply providing compliant answers.

**Table 3.** Dimensions and criteria for role-playing ability evaluation of a 5-point Likert scale^a^.

Competency dimension and performance metrics	Fulfillment criteria
The appeal of role-playing	
Anthropomorphism degree	Respond naturally and express reasonable human emotions and personality.
Diversity of expression	Dialogue behaviors and utterances are rich and diverse, avoiding repetitive expressions.
Role consistency	
Knowledge exposure	The response appropriately reflects the character’s background traits.
Knowledge hallucination	There is no fabrication of information unknown to the character and no violation of the character’s settings.
Stylistic consistency	The speaking style, wording habits, and tone descriptions conform to the patient’s personality and characteristics.
Conversation ability	
Fluency	The response is grammatically correct, and the expression is smooth.
Relevance	The response closely adheres to the conversation topic without deviation.
Logical consistency	In multiturn conversations, the responses are logically consistent and free of contradictions.

a The evaluation architecture incorporates 3 categorical elements: competency dimensions, performance metrics, and fulfillment criteria, collectively designed to quantify the system’s clinical interaction fidelity.

As for interaction efficiency and multiturn performance of the multiagent VP, practical efficiency was assessed by measuring average response time and token counts on a standard question set per dialogue turn. For multiturn performance, an autonomous physician agent was developed to simulate full consultations, guided by standard medical protocols and terminating upon complete information retrieval. Ground truth clinical information points in clinical case reports were annotated by 3 physicians, while GPT-4 served as an evaluator to identify these points in the dialogue history. An information accumulation curve was derived by tracking, for each turn, the cumulative percentage of disclosed information, defined as the ratio of cumulative information points identified to the total points in the ground truth. Metrics, including average dialogue turns, response length, and the accumulation curve, were analyzed to ensure the trajectory of clinical information disclosure aligned with the intended use requirements.

### Statistical Analysis

Statistical analyses were performed using Python (version 3.10; SciPy library). Continuous data were expressed as means (SD). Interrater reliability among experts was evaluated using the Gwet AC1 statistic. Given the matched-sample nature of the evaluations, overall performance differences across the 4 models for metrics, including accuracy, misleading rates, factual consistency, and role-playing ability, were assessed using the Friedman test. Subsequent post hoc pairwise comparisons were conducted using the 2-sided Wilcoxon signed-rank test. To rigorously control for multiple testing, the Benjamini-Hochberg false discovery rate procedure was applied to all pairwise *P* values, with an adjusted *P*<.05 considered statistically significant.

## Results

### Clinical Case Reports Repository Construction Results

Our MAS dynamically processes raw clinical case reports through the memory management agent, enabling real-time structuring without complex preprocessing. This design preserves original medical narratives while generating structured representations during operation, maintaining flexibility for simulating diverse clinical scenarios. The content of the original case report after segmentation and structuring by the framework is presented in Table 4, which illustrates the result of preserving information in accordance with minimal information units, in order to reduce interference between different pieces of information.

**Table 4.** Dictionary of clinical case reports after agent-based structured processing.

Module^a^ and unit^b^	Fact unit^c^
Chief complaint	
1	Pain in the right posterior tooth and facial swelling for 4 d
Present illness	
1	Spontaneous pain in the right posterior tooth during biting and gingival swelling pain for 4 d, with gradual pain relief but persistent facial swelling
2	No cold or heat sensitivity, night pain, or gingival pus discharge
3	Self-medicated with “metronidazole and ibuprofen” 3 d ago with some pain relief
Family history	
1	No significant history
Physical examination	
1	Atrial fibrillation and no recent checkups
Clinical findings	
1	Right facial swelling with slightly elevated skin temperature and normal skin color
2	46MO^d^: extensive caries, percussion pain (++), grade I mobility, and swollen buccal gingival sulcus without fluctuation
3	X-ray shows: crown radiolucency reaching pulp chamber, no root canal filling, and periapical radiolucency
4	48: impacted tooth
5	Poor oral hygiene with calculus ++, PD^e^: 4‐6 mm, detectable AL^f^
Diagnosis	
1	46: acute exacerbation of chronic periapical periodontitis
2	48: impacted tooth
3	Maxillary dentition defect
Management	
1	Loxoprofen sodium tablets 60 mg*36×1 box. Dosage: 60 mg PRN^g^ orally
2	Tinidazole tablets 0.5 g*8×2 boxes. Dosage: 0.50 g bid orally
3	Amoxicillin capsules 0.25 g*24×1 box. Dosage: 0.50 g tid orally

a Module refers to the designated segments into which a clinical case report is partitioned.

b Unit refers to the number of textual units into which the case information of each module is partitioned according to predefined rules. For example, the “present illness” section may be segmented into 4 discrete units of clinical information.

c Fact unit refers to the content obtained from the segmentation of specific case reports.

d 46MO: 46 mesio-occlusal.

e PD: pocket depth.

f AL: attachment loss.

g PRN: pro re nata.

### Evaluation Results

#### Evaluation Situation

Our output dataset comprised 650 physician-patient dialogues across 5 major medical specialties covering internal medicine, surgery, gynecology, pediatrics, and dentistry. Specifically, the expert evaluation covered 150 dialogue records of dental cases. The results of evaluation yielded several key findings, demonstrating the strong performance of the multiagent framework in simulating SPs. These results are presented across several main areas: factual consistency, accuracy and misleading rates, role-playing ability, and perceived educational utility.

#### Factual Consistency

**Figure 6.**
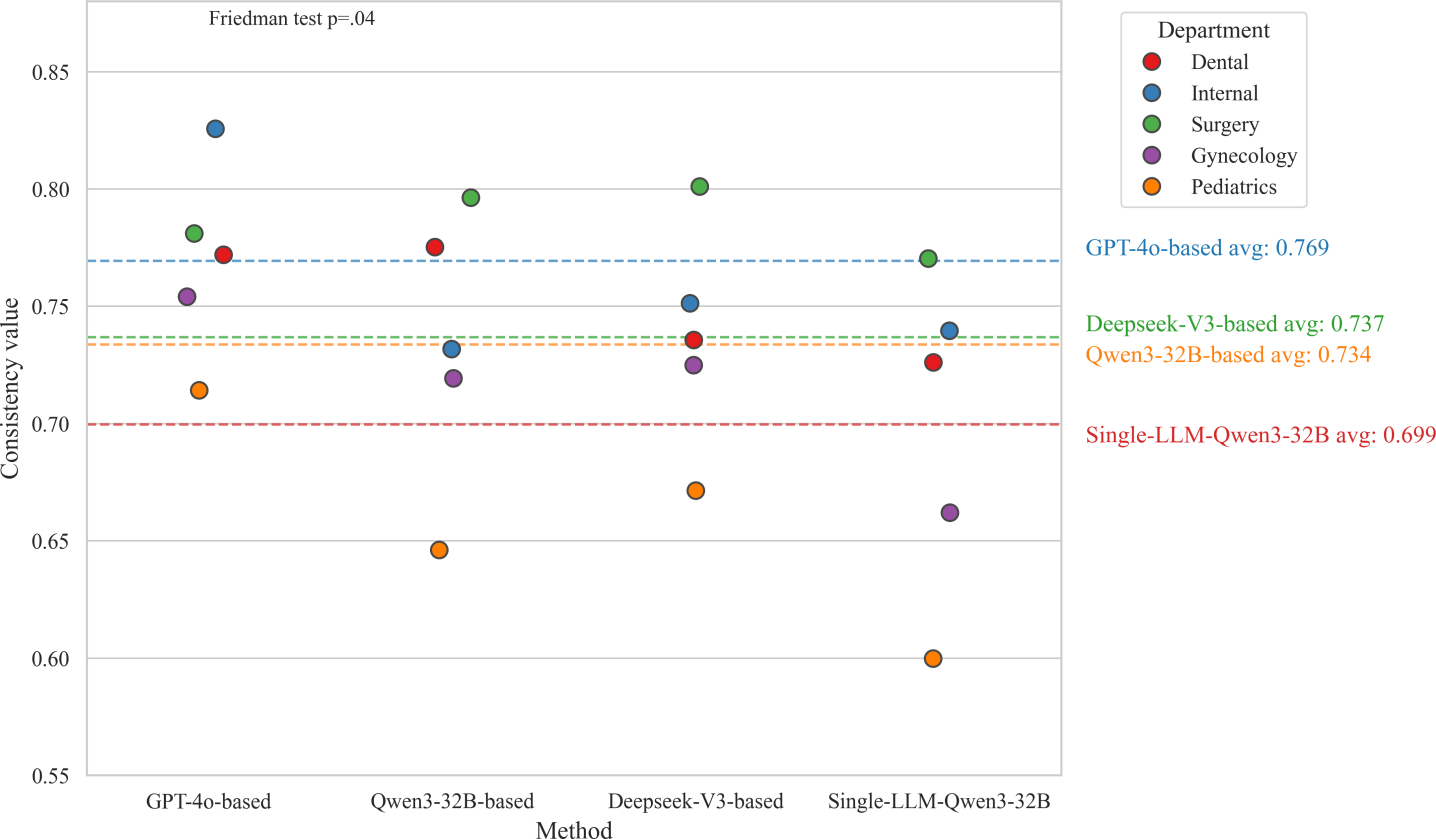
Factual consistency scores and mean values of multiagent framework with different base models. The dotted lines indicate the average (avg) score for each method.

Friedman test revealed a significant overall difference in factual consistency across methods (n=10; *χ*²_3_=13.0; *P*=.04). As Figure 6 illustrates, the GPT-4o–based approach performed best, leading across most departments with the highest mean score of 0.769 (SD 0.04), and significantly outperforming the single-LLM baseline (adjusted *P*=.03). While the Qwen3-32B multiagent framework achieved a higher mean factual consistency score of 0.734 (SD 0.06) compared to the single-LLM Qwen3-32B approach 0.699 (SD 0.07), this numerical improvement did not reach statistical significance (adjusted *P*=.33). Furthermore, DeepSeek-V3–based performance was similar to the Qwen3-32B–based framework. Across all methods, performance was consistently best in surgery and worst in pediatrics.

#### Accuracy and Misleading Rate

This study evaluated the accuracy of clinical information within dialogues generated from dental case reports. All methods demonstrated high proficiency, with accuracy rates exceeding 94%. Statistical analysis revealed a significant overall performance variance among the models (*χ*²_3_=12.0; *P*=.007; Table 5). The DeepSeek-V3–based framework and the single-LLM baseline emerged as the top-performing approaches (mean 97.44, SD 1.24). Conversely, the GPT-4o–based framework recorded the lowest relative accuracy and was significantly outperformed by both the single-LLM baseline (adjusted *P*=.03) and the DeepSeek-V3 framework (adjusted *P*=.03). Notably, despite achieving the highest mean accuracy, the single-LLM baseline did not demonstrate a statistically significant advantage over the Qwen3-32B model (adjusted *P*=.17). This comparable performance indicates that the multiagent framework did not yield a significant difference in accuracy over the single-LLM approach.

**Table 5.** Accuracy and misleading rates of different methods (N=8)^a^^,^^b^.

Method	Accuracy (%), mean (SD)	Misleading (%), mean (SD)
Single-LLM-Qwen3-32B	97.44 (1.24)^A^	4.72 (1.53)^A^
DeepSeek-V3–based	97.44 (1.24)^A^	1.39 (0.87)^B^
Qwen3-32B–based	96.15 (2.05)^A,B^	1.28 (1.20)^B^
GPT-4o–based	94.87 (1.82)^B^	2.56 (1.01)^A,B^

a Interrater reliability analysis showed high consensus (Gwet AC1: 0.93 for accuracy and 0.96 for misleading rate).

b Superscript letters (Aand B) across rows denote significant pairwise differences (adjusted *P*<.05). Models sharing the same letter do not differ significantly.

For noncase report–based information, all methods demonstrated misleading rates under 5%, again showing a significant overall difference (*χ*²_3_=15.73; *P*=.001; Table 5). The single-LLM Qwen3-32B baseline showed significantly higher misleading rates than the multiagent frameworks based on Qwen3-32B (adjusted *P*=.003) and DeepSeek-V3 (adjusted *P*=.004). Differences between other models regarding misleading rates were not significant (adjusted *P*≥.11). The multiagent framework may reduce hallucinations by retrieving only case-relevant information.

#### Role-Playing Ability and Perceived Educational Utility Under Standard Conditions

**Figure 7.**
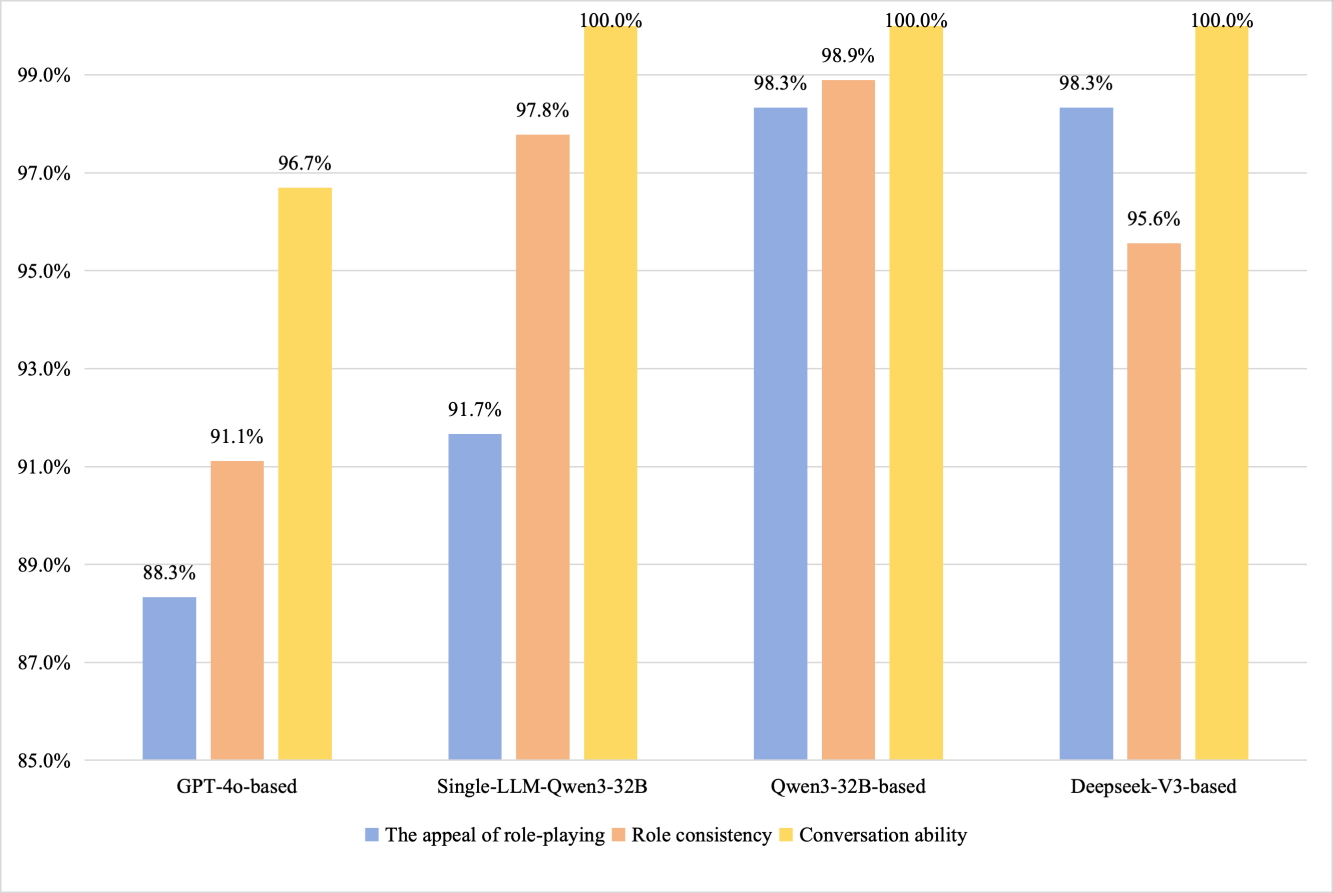
A detailed analysis of score distributions across the 3 evaluation dimensions evaluated by physicians.

For the role-playing competency assessment based on a standard inquiry test set, experts scored 8 dental case dialogues using an 8-item scale across 3 dimensions. The detailed scores for each dimension are presented in Table 6. Overall, a statistically significant difference in total role-playing competency scores among the 4 models was observed (*χ*²_3_=16.6; *P*<.001). The results demonstrated that the Qwen3-32B–based framework achieved a significantly higher total score (mean 39.67, SD 0.71) compared to the GPT-4o–based framework (*P*=.008) and the single-LLM baseline (*P*=.04). Differences between DeepSeek-V3–based and either Qwen3-32B–based (*P*=.31) or the single-LLM Qwen3-32B (*P*=.48) were not statistically significant. A detailed analysis of score distributions across the 3 evaluation dimensions was conducted in Figure 7. Regarding role attractiveness, the role-playing degree and expressive diversity exhibited by the VPs were the primary focus of assessment. Examples of VP responses from different methods are presented in Table 7. The results indicated that Qwen3-32B–based agents and DeepSeek-V3–based agents achieved 98.3% (9.83/10) of the maximum score for the appeal of role-playing, demonstrating diverse human-like characteristics, natural interaction, and appropriate emotion. The GPT-4o–based system scored 88.3% (SD 0.07; 8.83/10), exhibiting a more direct, rational style with occasional neglect of patient personality. The single-LLM baseline obtained a score between these top-performing models and the GPT-4o–based system.

**Table 6.** Detailed scores for the 8 consistency issues in the dental cases, as evaluated by physicians using the standardized scale (N=8)^a^.

Methods	The appeal of role-playing, mean		Role consistency, mean			Conversation ability, mean			Total score
	AD^b^ (Krippendorff α=0.67)	DE^c^ (Krippendorff α=0.66)	KE^d^ (Krippendorff α=0.72)	KH^e^ (Krippendorff α=0.77)	SC^f^ (Krippendorff α=0.73)	Flu.^g^ (Krippendorff α=0.87)	Rel.^h^ (Krippendorff α=0.87)	LS^i^ (Krippendorff α=0.87^j^)	
GPT-4o–based	4.33	4.50	4.50	4.50	4.67	4.83	5.00^k^^,^^l^	4.67	37.33^C^
Single-LLM-Qwen3-32B	4.67	4.50	5.00^k^^,^^l^	4.67	5.00^k^^,^^l^	5.00^k^^,^^l^	5.00^k^^,^^l^	5.00^k^^,^^l^	38.83^B^
Qwen3-32B–based	4.83	5.00^k^^,^^l^	5.00^k^^,^^l^	4.83^k^	5.00^k^^,^^l^	5.00^k^^,^^l^	5.00^k^^,^^l^	5.00^k^^,^^l^	39.67^A,k,l^
DeepSeek-V3–based	5.00^k^^,^^l^	4.83	4.67	4.83^k^	4.83	5.00^k^^,^^l^	5.00^k^^,^^l^	5.00^k^^,^^l^	39.17^A,B^

a Superscript letters (A, B, and C) alongside the total scores denote significant pairwise differences (adjusted *P*<.05). Models sharing the same letter do not differ significantly.

b AD: anthropomorphism degree.

c DE: diversity of expression.

d KE: knowledge exposure.

e KH: knowledge hallucination.

f SC: stylistic consistency.

g Flu.: fluency.

h Rel.: relevance.

i LS: logical consistency.

j Interrater reliability indicating strong expert consensus.

k Optimal values for each indicator.

l Optimal values

**Table 7.** Example comparison results of dialogue transcripts between multiagent frameworks with different base models and single-large language model approaches.

Method	Communication record Example: Is there heavy bleeding from your gums? Does it stop by itself?
GPT-4o–based	The bleeding is not heavy and usually stops on its own.
Qwen3-32B–based	The bleeding isn’t heavy, just some blood streaks when brushing, and occasionally a little bleeding even with light contact, but it stops on its own and resolves quickly. I’m a bit concerned, could this be some kind of inflammation?
Single-LLM-Qwen3-32B	It’s not much bleeding, just a little bit. Sometimes it looks slightly red right after brushing, but it stops on its own after a while.
DeepSeek-V3–based	The bleeding isn’t heavy, just some pink streaks on my toothbrush that go away after rinsing a couple times. But it keeps oozing like this, and I’m worried. Could there be a risk it might suddenly stop clotting one day?

Regarding the role consistency on the standard inquiry test set, Qwen3-32B–based agents achieved 98.9% (SD 0.02; 14.83/15), effectively portraying character settings. In contrast, GPT-4o–based agents scored 91.1% (SD 0.01; 13.67/15), exhibiting fabrication or neglect of character details and limited background representation. Regarding conversational competence, all methods achieved scores exceeding 96.7% (SD 0.02%; 14.5/15), closely adhering to the defined criteria of grammatical accuracy, fluency of expression, logical consistency across multiple turns, thematic relevance, and absence of deviation. This suggests that current LLMs can effectively simulate SP dialogues.

**Figure 8.**
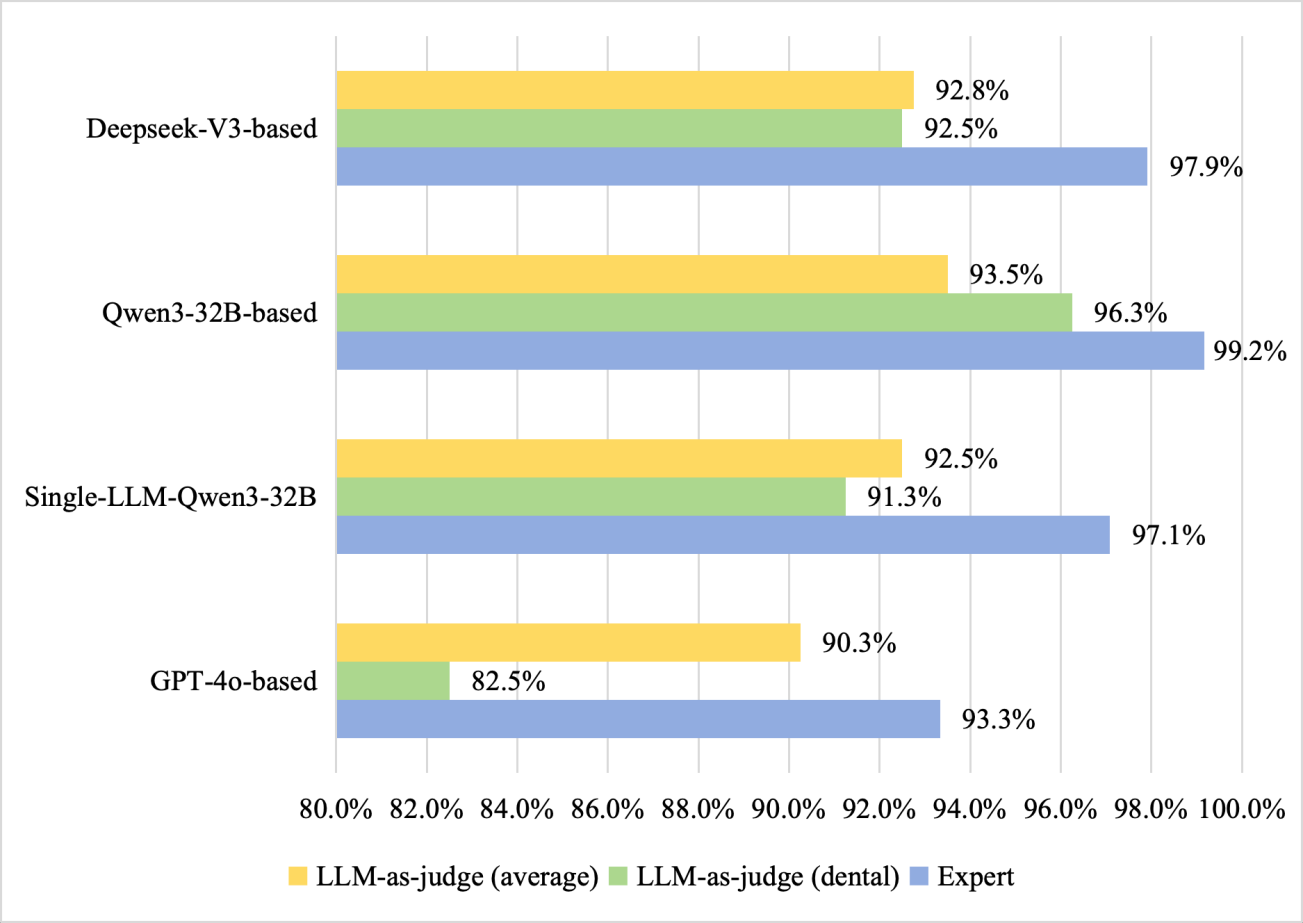
Total role-playing score performance of virtual patient by physicians (dental), LLM-as-judge (dental), and the average scores of LLM-as-judge across 5 clinical specialties. LLM: large language model.

**Figure 9.**
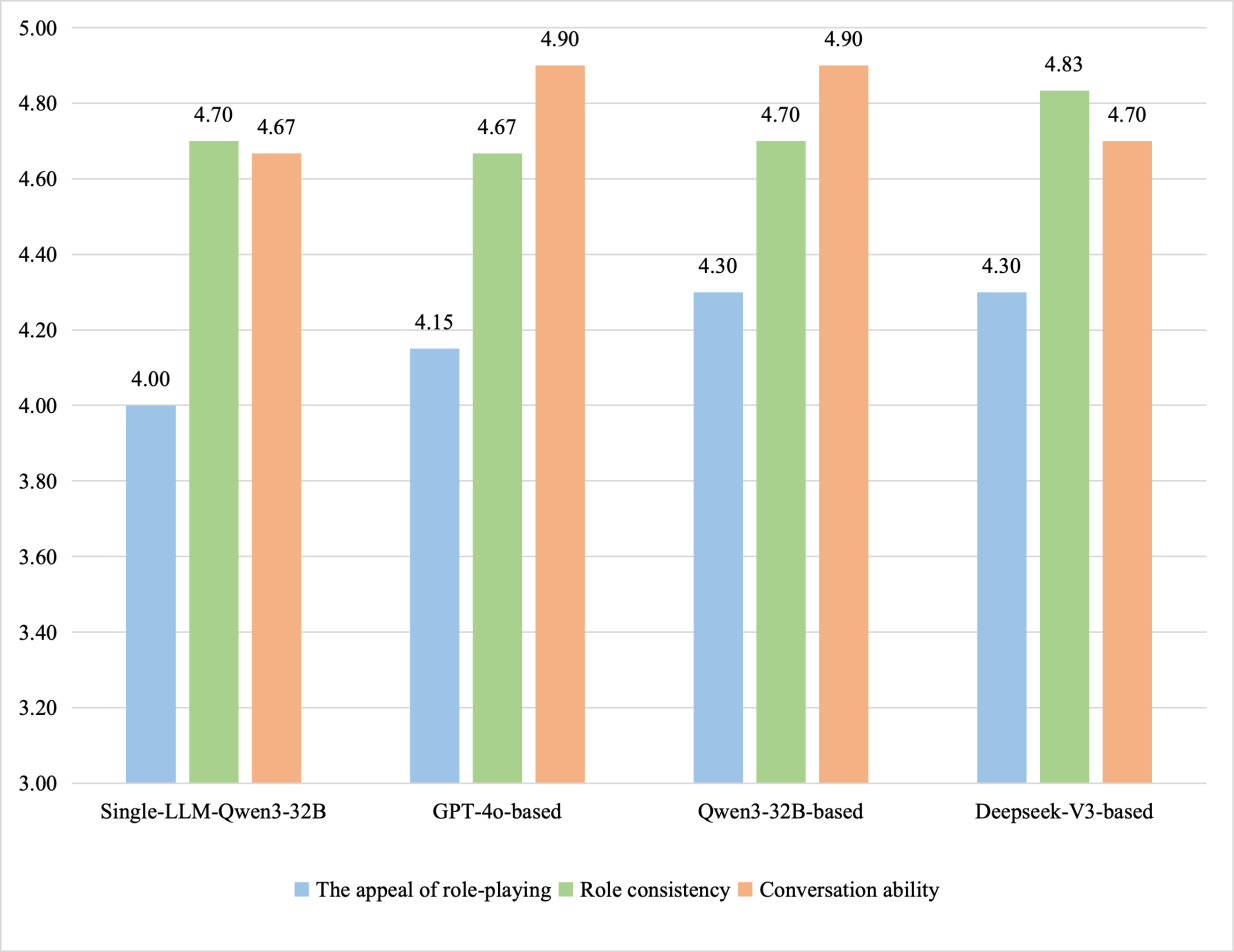
Role-playing competency scores of virtual patients across 5 specialties during simulated dialogues evaluated by GPT-4.

Meanwhile, comparative analysis revealed that the LLM-as-judge scores, assessed by GPT-4 using standardized rating scales, were systematically lower than expert ratings. The overall scoring trends demonstrated substantial alignment between both evaluation methods, as shown in Figure 8. Figure 9 presents the LLM-as-judge evaluation results across all 5 clinical specialties on average. Both Qwen3-32B and DeepSeek-V3 achieved the highest scores in 2 distinct dimensions, consistent with trends observed in leading performance rankings.

In terms of perceived educational utility, the multiagent framework based on the DeepSeek-V3 and Qwen3-32B received full scores from medical physicians under standard inquiry conditions, as illustrated in Table 8. This indicates their applicability for physician-patient communication training within the medical education process.

**Table 8.** Perceived educational utility score for the standard inquiry test set assessing the acceptability level of applying virtual patient in real physician-patient communication training^a^.

Methods	Score, mean (SD)
GPT-4o–based	4.50 (0.71)
Single-LLM-Qwen3-32B	4.83 (0.24)
Qwen3-32B–based	5.00 (0.00)
DeepSeek-V3–based	5.00 (0.00)

a The interrater reliability (Krippendorff α) for this metric was 0.75, indicating strong expert consensus. Statistical significance testing was not performed, as it is intended to provide a holistic perspective from clinicians on the overall educational value of the system.

#### Exploratory Analysis of Role-Playing Ability Under Low-Quality Inquiry

**Figure 10.**
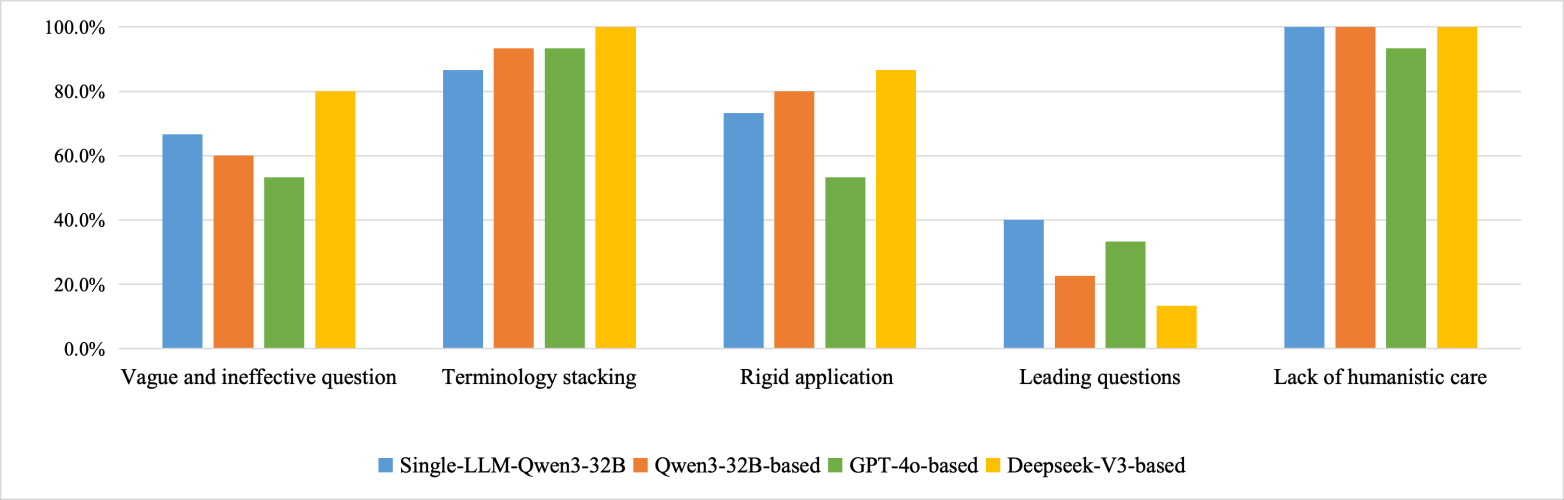
Performance scores of virtual patients in response to low-quality medical inquiries. Interrater reliability (Gwet AC1) for each dimension is as follows: dimension VI (AC1=0.66), dimension Ts (AC1=0.84), dimension Ra (AC1=0.62), dimension Lq (AC1=0.63), and dimension LHC (AC1=0.96), confirming great agreement among experts on these binary metrics.

As for the exploratory analysis of the low-quality inquiry test set, our multiagent framework demonstrated limited advantages in specific scenarios, effectively identifying low-quality medical inquiries, such as terminology stacking and rigid application, and responding according to predefined role configurations, as shown in Figure 10. However, the framework exhibits insufficient discriminative power in handling other types of low-quality inquiries. Specifically, regarding the lack of humanistic care, scores remained consistently high across different methods without significant differences. In contrast, performance was notably inferior when addressing leading questions, in which the model demonstrated a tendency toward overreaction.

#### Scalability

**Figure 11.**
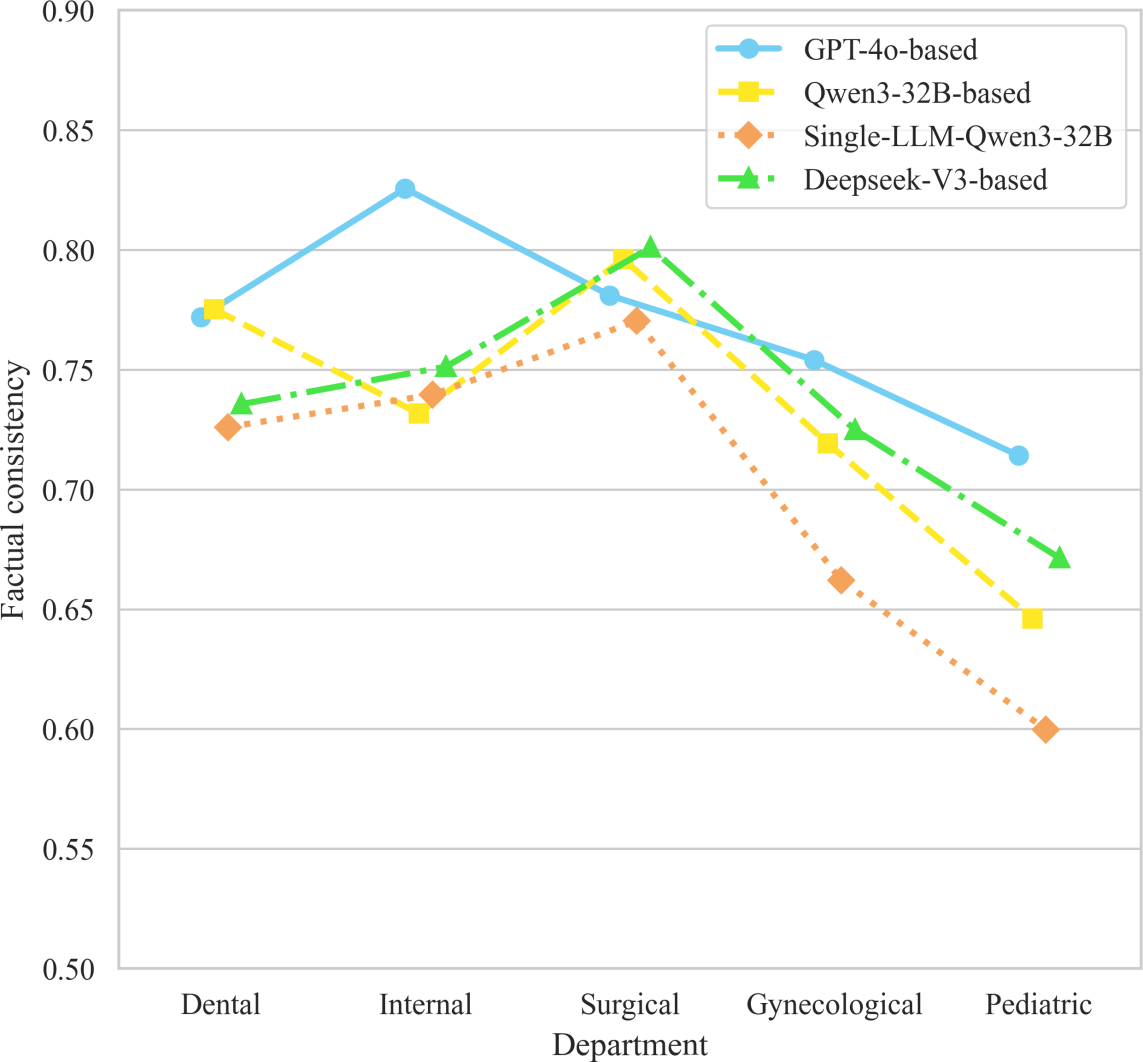
Corresponding score distribution curves of factual consistency results for multiagent frameworks with different specialty case reports and different base models.

We preliminarily evaluated the scalability of our framework by assessing its factual consistency and role-playing performance, using LLM-as-judge evaluations as a preliminary exploratory approach. The line graph in Figure 11 illustrates the performance variations of each method across case reports from different clinical departments. Trend analysis reveals that, with the exception of the GPT-4o–based framework, the case reports from different departments had a consistent impact on the performance trends of the other methods. The factual consistency scores for pediatrics were notably lower than those of the other 4 departments, indicating that case reports from this specialty may have a substantial impact on the VP’s responses. The other 4 departments exhibited similar scoring ranges, with our method demonstrating stable fluctuations within a high-scoring interval (0.7‐0.85), suggesting good generalizability and broad applicability across diverse case reports.

Analysis of the CV revealed that the GPT-4o–based framework (CV=4.7%) exhibited the highest stability, while single-LLM Qwen3-32B framework (CV=8.7%) showed slightly greater variability. As illustrated in Table 9, the overall model performance was minimally affected by clinical departments. However, factual consistency scores for single-LLM-Qwen3-32B framework varied more substantially across departments, indicating lower scalability compared to the multiagent framework.

**Table 9.** Comparison of the coefficient of variation (CV) across different methods.

Methods	CV, %
GPT-4o–based	4.7
Qwen3-32B–based	7.1
Single-LLM-Qwen3-32B	8.7
DeepSeek-V3–based	5.7

**Figure 12.**
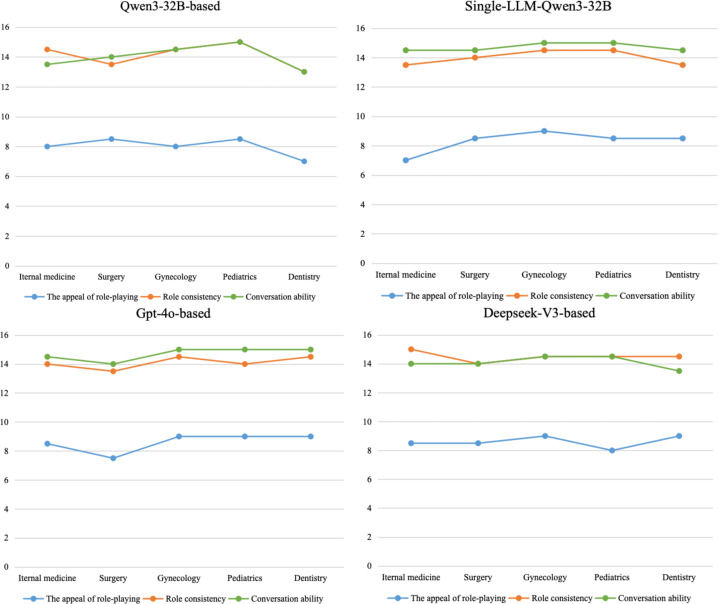
Performance scores of role-playing competencies across specialties based on GPT-4 automated evaluation.

The LLM-as-judge evaluation across different specialty-specific VP dialogues revealed that our framework demonstrated stable cross-specialty performance , with limited score variations in the appeal of role-playing (*Δ*<2), outperforming single-LLM baseline’s greater variability, as shown in Figure 12. DeepSeek-V3 based showed minimal fluctuations in role attractiveness (*Δ*=1). These findings suggest that while our framework enhances scalability in the appeal of role-playing in certain clinical departments, the performance of the base model should be considered as a critical factor during deployment.

#### Interaction Efficiency and Multiturn Dialogue Performance

**Figure 13.**
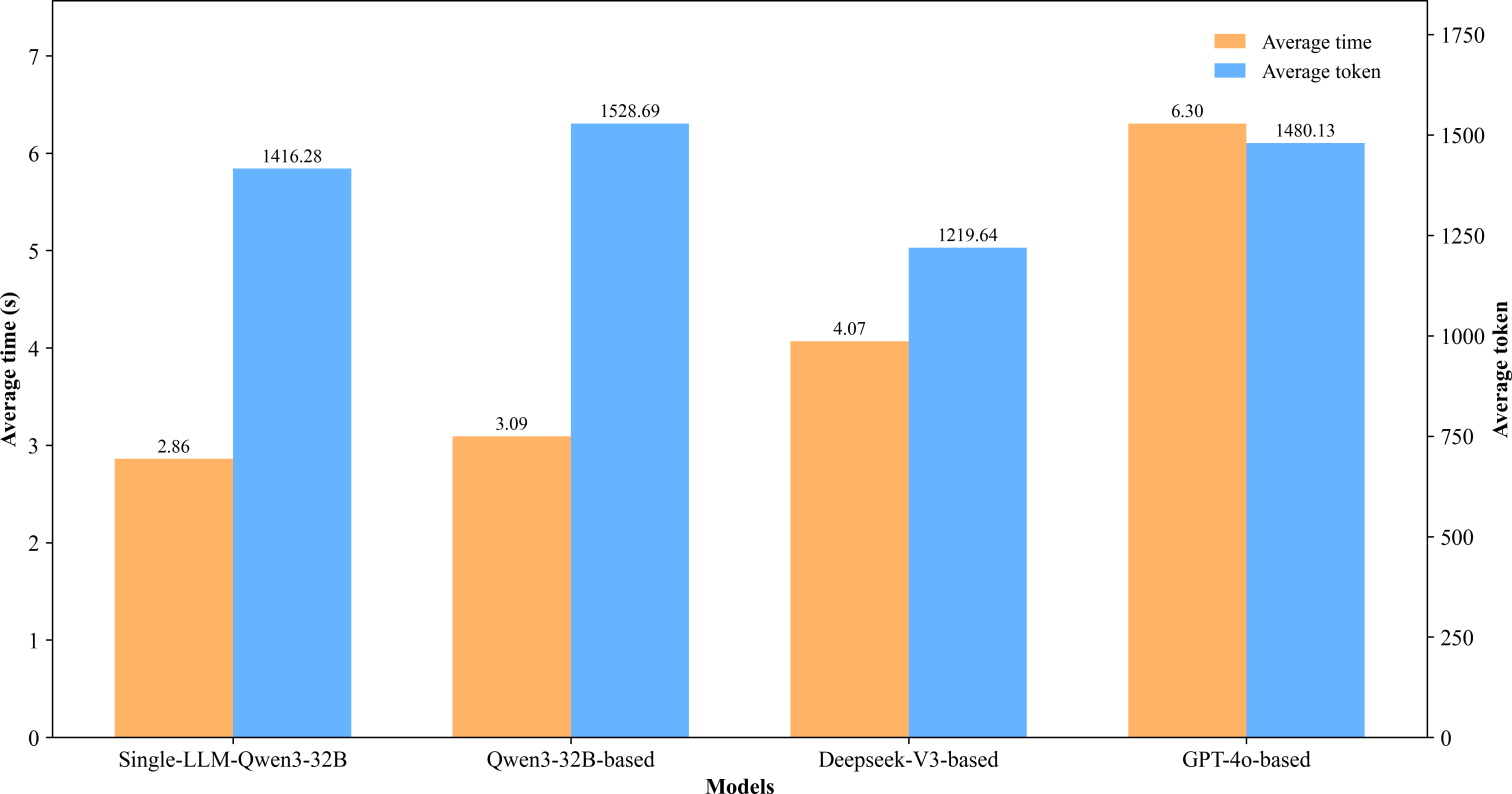
Interaction efficiency evaluation based on average time and average token.

Regarding interaction efficiency, the Qwen3-32B–based multiagent VP achieved the fastest response rate among the 3 backbone models, slightly longer than the single-LLM Qwen3-32B. For the Qwen3-32B–based VP, the average latency of approximately 3 seconds fell within an acceptable range for user interaction, as shown in Figure 13. In terms of resource usage, DeepSeek-V3–based VP proved the most efficient (mean 1219.64 tokens (SD 130.95)), whereas the Qwen3-32B–based VP consumed the most. Although the Qwen3-32B–based VP used more tokens than the single-LLM baseline, its consumption remained stable across sessions, contrasting with the high variance observed in the single-LLM baseline.

**Figure 14.**
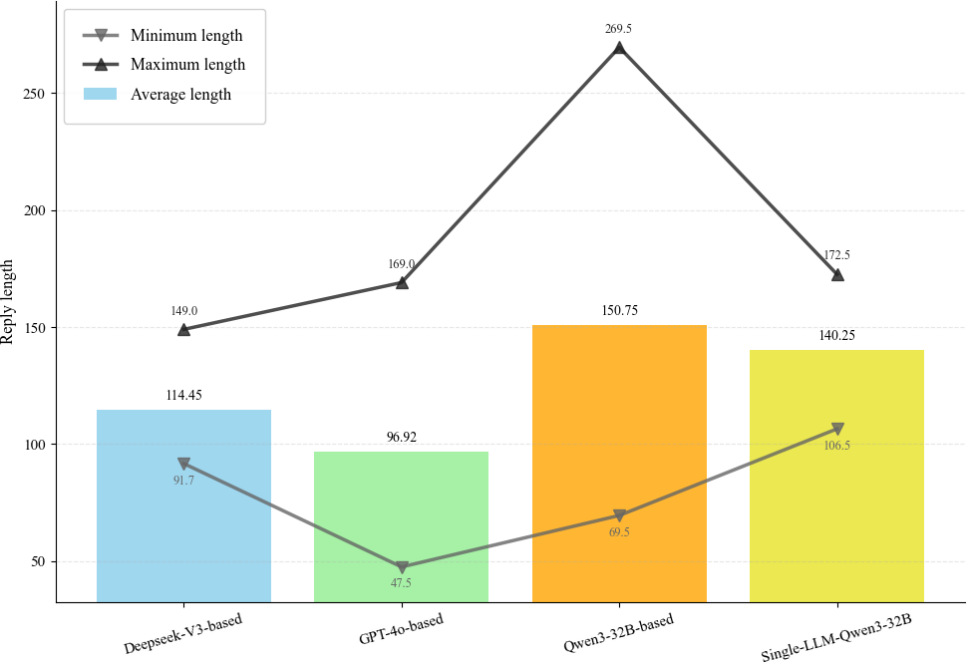
Statistical distribution of reply lengths across different methods, illustrating the average length alongside minimum and maximum values.

**Figure 15.**
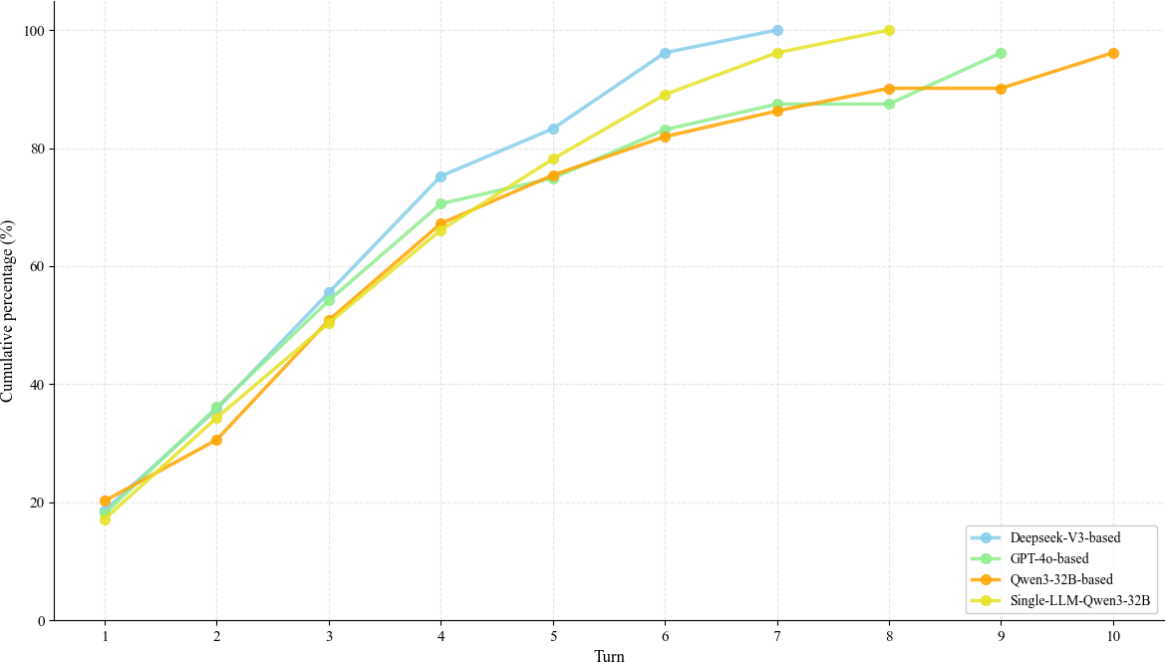
Information accumulation curve.

For multiturn performance, the Qwen3-32B–based VP achieved the highest engagement and average response length, averaging 13 turns according to Table 10 and Figure 14. Regarding the dynamics of the interaction, the analysis of information accumulation in Figure 15 reveals distinct differences in information pacing. Specifically, the baselines built on GPT-4o and Qwen3-32B exhibited an information accumulation rate trend characterized by an initial rise, subsequent fall, and final rise. In contrast, DeepSeek-V3 and the single-LLM baseline approach showed a trajectory, featuring a rapid initial rise followed by a decline. These diverging patterns highlight variations in information flow during the dialogue. The rapid early accumulation observed in the latter group suggests a more rapid information release strategy, which could indicate either higher efficiency or a tendency toward excessive disclosure in early turns. Ultimately, this indicates that sustainable multiturn dialogue relies on a balanced information release rhythm, and avoiding early information saturation might be more conducive to dialogue stability.

**Table 10.** Average conversation turns by different methods

Method	Values, mean (SD)
DeepSeek-AI–based	6.333 (0.882)
GPT-4o–based	11.5 (4.950)
Qwen3-32B–based	13 (4.243)
Single-LLM-Qwen3-32B	7.5 (1.732)

### Multiagent Workflow Instance

**Figure 16.**
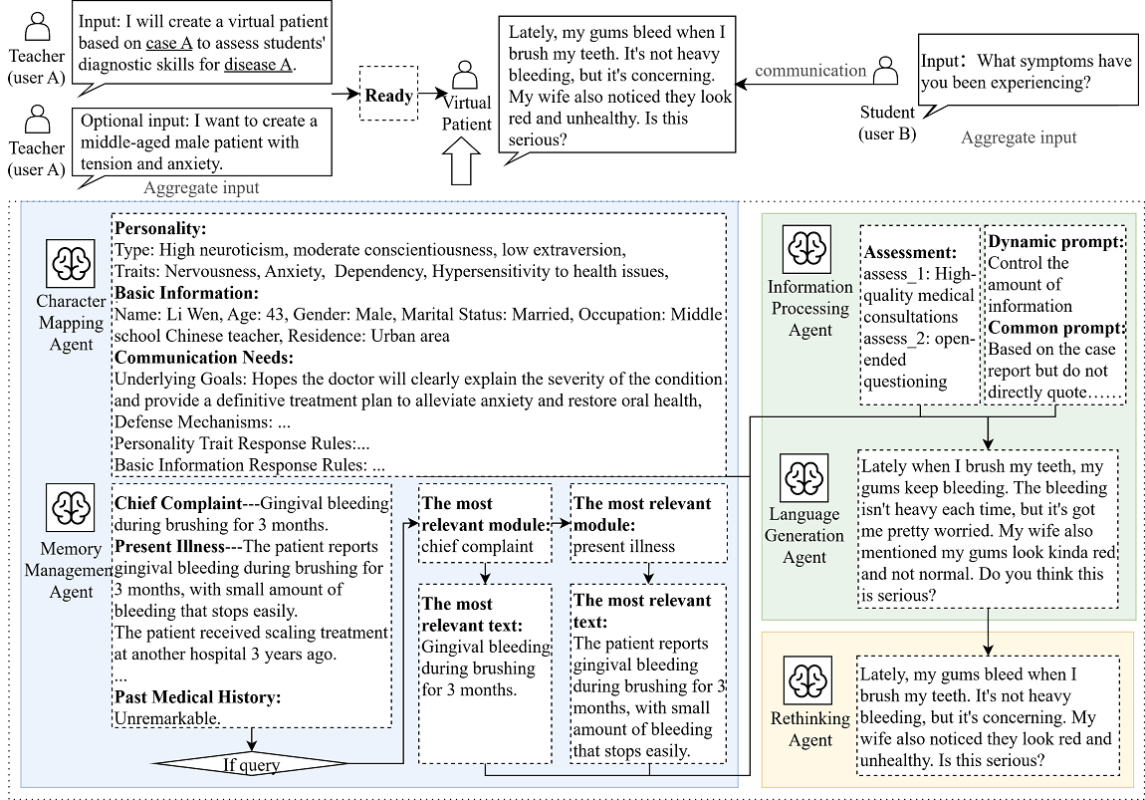
An input and output example with the multiagent framework.

Figure 16 exemplifies the multiagent workflow for VP construction and response generation. Upon case confirmation by medical educators, agents collaborate to develop a tailored VP based on specified requirements. These agents construct patient-specific information aligning with the case’s core features. The example illustrates a middle-aged male patient with clinical anxiety, cautiousness, dependency, and health concern. Concurrently, the information processing agent segments the case report and stores clinical data for real-time retrieval during student interactions.

When a simulated student inquiry asks, for instance, “Where have you been feeling uncomfortable recently?,” the VP response framework activates. The system classifies this query as high quality and open ended, which triggers the selection of an optimized prompt to generate a relevant response. Simultaneously, the system uses clinical reasoning to connect the query to the most relevant case modules, such as the patient’s chief complaint and present illness. Through structured traversal of minimal information units within these modules, the agent precisely identifies and retrieves the most clinically relevant data elements for response generation.

The response generation agent generates replies based on information input from other subagents. The reflection agent performs final-stage quality control by verifying response compliance with SP protocols. Responses satisfying all clinical and educational standards are directly delivered to users, while noncompliant responses undergo targeted modification to rectify specific deviations before being output.

## Discussion

### Principal Findings

Our study successfully developed a multiagent framework for SP simulation and evaluated its performance against single-LLM baseline setups. The principal findings reveal that the multiagent framework’s structured design and the resulting disparity in prompt depth significantly improved VP simulation quality by enhancing factual consistency, reducing misleading responses, increasing role-playing expressivity, and demonstrating stable performance in terms of interaction efficiency and multiturn dialogues.

The high accuracy rate underscores the framework’s clinical correctness, while factual consistency reflects semantic alignment with case reports. Together, these complementary metrics indicate that the VP responses are both semantically grounded and clinically reliable. While the GPT-4o–based framework exhibited the highest factual consistency, it yielded the lowest accuracy. This implies a specific weakness in pinpointing targeted consultation information despite generating fluent, context-similar text. In contrast, Qwen3-32B–based and DeepSeek-V3–based achieved peak accuracy scores. Despite yielding a higher, albeit nonsignificant, medical accuracy compared to Qwen3-32B–based, the single-LLM baseline exhibited significantly higher misleading rates than both the Qwen3-32B–based and DeepSeek-V3–based multiagent frameworks. These findings suggest that the absence of decomposed reasoning increases the susceptibility to hallucinated content.

Our framework reinforces patient characteristics by reasoning deeply about superficial traits, which helps create richer personality profiles and guides the LLM’s persona performance, aligning with role-playing agent research on role identity activation [44]. Evaluations showed that in role-playing capability and perceived educational utility, the Qwen3-32B framework performed comparably to the DeepSeek-V3 implementation, with both significantly outperforming the GPT-4o–based one. It is noteworthy that our multiagent framework achieved a higher score in role-playing than the single-LLM baseline on the standard inquiry test set. This observation can be attributed to the deep role-response reasoning implemented in our subagents. Physicians also strongly endorsed our framework’s educational utility with a high score. The inverse relationship between factual consistency and role-playing implies that maximizing similarity to clinical facts is a limited proxy for realistic simulation, as authentic patients rarely speak with such medical precision. Thus, factual consistency should serve as a necessary baseline for clinical correctness rather than a target to be maximized in isolation, avoiding rigid alignment that hinders naturalistic expression.

Regarding the exploratory analysis of the low-quality inquiry test set, specifically for terminology stacking and rigid application, the results indicate that the framework adheres to character constraints more effectively than the single-LLM baseline, yet exhibits limited discriminative behavioral dynamics when challenged by subtle cues such as leading questions. Overall, the results demonstrate that our framework enhances the VP’s capability to maintain its persona compared to the single-LLM baseline.

As for factual consistency across diverse clinical departments, the framework maintained scores within the 0.7 to 0.85 range except pediatrics, with a lower CV than the single-LLM baseline, suggesting improved stability and potential for rapid construction without complex preprocessing [31,45]. The drop in pediatrics likely resulted from the simulation of the child patients who typically have limited communicative ability. This indicates that role definitions in specific departments require adjustment, such as simulating a guardian to better support realistic medical education. In terms of role-playing ability, preliminary LLM-as-judge evaluations suggested consistent cross-specialty performance, with limited score variations (*Δ*<2) observed in the appeal of role-playing. However, despite expert review of the generated test set, the use of GPT-4 for both creating the test set and the subsequent evaluation introduces a risk of self-preference bias or circular validation. Therefore, these role-playing metrics serve primarily as an exploratory reference, and definitive verification necessitates further validation by human specialists.

MASs face significant challenges regarding time and cost efficiency in practical applications. While our multiagent framework based on Qwen3-32B exhibited a higher average response time and higher average token consumption compared with the single-LLM baseline, its performance remained within acceptable limits, demonstrating that the efficiency of our framework was acceptable. Regarding multiturn dialogue, the average number of dialogue turns and the steady growth of accumulated information points across turns reflect the gradual information release pace essential to simulated clinical settings, highlighting the framework’s potential for application.

Overall, our proposed framework demonstrated superior performance compared to the single-LLM baseline across most evaluation metrics, while maintaining an acceptable efficiency profile. Collectively, these findings highlight the importance of optimizing the trade-off between time efficiency and response quality when selecting model configurations for real-world deployment. While this study prioritizes real-time interaction fidelity, we acknowledge that alternative deployment settings may yield different trade-offs between reasoning depth and latency. In particular, a single LLM with reasoning enabled could offer stronger analytical performance in offline or low–time-sensitivity educational scenarios, such as post hoc case analysis or self-paced learning. However, our preliminary evaluations indicate that enabling reasoning in large-scale models introduces substantial response latency and variability, which undermines conversational realism in interactive patient simulations. Consequently, the current framework adopts multiagent coordination under constrained inference settings as a more practical solution for real-time clinical education, while future work may explore adaptive reasoning strategies that dynamically balance responsiveness and analytical depth across diverse learning contexts.

### Comparison With Prior Work

To further evaluate the performance of our framework, we compared its evaluation results with those of existing MASs designed for role-playing. Specifically, in terms of factual consistency, we compared our framework with an existing system Mediq [46]. Our GPT-4o–based framework achieved a score of 82.6% in medical dialogues, which is higher than Mediq’s direct (55.9%) and instruct (62.8%) variants but lower than its fact-select approach. The difference may stem from our emphasis on patient personality, which can reduce textual similarity to the original case reports. Our framework also achieved significantly higher average scores in conversational ability and the appeal of role-playing compared to top-performing GPT-4–based implementations in the CharacterEval benchmark [43]. Although role consistency was slightly lower, likely because dialogues did not always require explicit trait expression, expert evaluations confirmed consistency with LLM-as-judge rankings, validating the Qwen3-32B–based framework’s robust role-playing capabilities, corroborating prior role-playing agent studies on role identity activation [44].

### Educational Implications for Medical Training

From an educational perspective, the proposed multiagent VP framework offers several practical implications for clinical communication training, exemplifying the pivotal role of generative AI in advancing medical education [47,48]. First, by maintaining stable patient personas while handling low-quality or incomplete inquiries, which have been noted in [49] to trigger hallucinations and response instability, the framework supports repeated, self-directed practice for medical students, particularly in early-stage communication skill acquisition. Second, the controllable and scalable nature of the system allows instructors to rapidly generate diverse SP scenarios without the logistical constraints associated with human SP recruitment and training [50]. Third, the observed reduction in misleading responses compared with the single-LLM baseline is especially relevant for formative educational settings, where inaccurate patient feedback may negatively reinforce incorrect clinical reasoning. As Wen et al [51] noted, single generative language models tend to entail inherent misleading risks in clinical-related interactions. Such behaviors directly align with the concern that misleading feedback from single LLMs may reinforce wrong clinical reasoning in formative education. Collectively, these findings suggest that the multiagent VP framework may serve as a complementary educational tool alongside traditional SP-based training, particularly in resource-limited or large-scale teaching contexts.

### Limitations

This study has several limitations. First, our evaluation focused on the overall performance of the multiagent framework rather than the isolated effectiveness of individual subagents. While this design demonstrates the advantages of collaborative agent-based modeling, it does not fully disentangle the specific contributions of each component, such as the memory management agent or the character mapping agent. Importantly, the comparison between the multiagent framework and the single-LLM baseline was not fully matched in terms of prompt depth. As shown in Table 1 in Multimedia Appendix 1, the single-LLM baseline relied primarily on teacher-provided patient settings, whereas the multiagent framework used a dedicated character mapping agent to expand these inputs into a richer personality profile, including behavioral tendencies, response thresholds, and defense mechanisms. This difference introduces a potential confounding factor, as the observed performance gap may reflect not only architectural advantages of multiagent coordination but also differences in prompt engineering depth. Consequently, future research should conduct more controlled ablation studies to isolate the effects of individual subagents and systematically evaluate the impact of prompt complexity, thereby disentangling architectural contributions from prompt design effects.

Second, although our study conducted a comprehensive evaluation, it did not include a prospective validation in real-world clinical settings. The assessment relied primarily on controlled scripts and LLM-as-a-judge methods, which may not fully capture the complexity of authentic student-patient interactions. While preliminary expert-based evaluation suggested high perceived educational utility, the long-term effectiveness and pedagogical impact of the framework remain uncertain. Further studies should incorporate randomized controlled trials in real teaching environments to rigorously assess whether the proposed system improves clinical communication skills compared with SPs or conventional training approaches.

Third, in addition to role-playing ability and multiturn dialogue evaluation, we assessed the response quality by incorporating specific natural language metrics, mirroring the essential SP function of conveying patient history and information during clinical dialogues. However, it is crucial to acknowledge the applicability boundary of these metrics that higher factual accuracy does not intrinsically mean greater patient simulation performance, as authentic patient behavior inherently involves forgetfulness, logical gaps, and potential fabrication. Future research should explore more comprehensive evaluation frameworks that better reflect real-world communication dynamics.

Finally, this study relied exclusively on Chinese clinical databases and expert evaluators. This linguistic homogeneity implies that the observed performance may stem from the base model’s alignment with specific training data rather than inherent architectural advantages. To ensure the framework’s generalizability and linguistic robustness, future validation on English and other multilingual datasets is essential.

### Conclusions

This study demonstrates that our multiagent framework, leveraging LLMs, provides a feasible method for SPs simulation. It facilitates natural and acceptable language-based interactions between VPs and users. In the context of medical education, this approach supports a promising pathway toward the development of scalable and effective communication training. Our multiagent framework demonstrated high factual consistency, response accuracy, and role-playing ability, while also demonstrating stable performance in terms of interaction efficiency and multiturn dialogues. This design overcomes the limitations of single-LLM baseline in medical education role-playing, effectively mitigating hallucinations and significantly reducing the rate of misinformation. Furthermore, our framework addresses limitations in case scenario updates and customization often found in existing multiagent VP systems, exhibiting high flexibility and scalability in certain clinical departments. This design shows promise in providing training-oriented support that complements theoretical coursework. Future studies should further explore its long-term efficacy and broader applicability in authentic medical teaching environments.

## Supplementary material

10.2196/84747 Multimedia Appendix 1 Prompts.

## Abbreviations

CV: coefficient of variation
LLM: large language model
MAS: multiagent system
SP: standardized patient
VP: virtual patient
